# Interaction between Long-Term Potentiation and Depression in CA1 Synapses: Temporal Constrains, Functional Compartmentalization and Protein Synthesis

**DOI:** 10.1371/journal.pone.0029865

**Published:** 2012-01-17

**Authors:** Alice Pavlowsky, Juan Marcos Alarcon

**Affiliations:** Department of Pathology, The Robert F. Furchgott Center for Neural and Behavioral Science, State University of New York, Downstate Medical Center, Brooklyn, New York, United States of America; Harvard University, United States of America

## Abstract

Information arriving at a neuron via anatomically defined pathways undergoes spatial and temporal encoding. A proposed mechanism by which temporally and spatially segregated information is encoded at the cellular level is based on the interactive properties of synapses located within and across functional dendritic compartments. We examined cooperative and interfering interactions between long-term synaptic potentiation (LTP) and depression (LTD), two forms of synaptic plasticity thought to be key in the encoding of information in the brain. Two approaches were used in CA1 pyramidal neurons of the mouse hippocampus: (1) induction of LTP and LTD in two separate synaptic pathways within the same apical dendritic compartment and across the basal and apical dendritic compartments; (2) induction of LTP and LTD separated by various time intervals (0–90 min). Expression of LTP/LTD interactions was spatially and temporally regulated. While they were largely restricted within the same dendritic compartment (compartmentalized), the nature of the interaction (cooperation or interference) depended on the time interval between inductions. New protein synthesis was found to regulate the expression of the LTP/LTD interference. We speculate that mechanisms for compartmentalization and protein synthesis confer the spatial and temporal modulation by which neurons encode multiplex information in plastic synapses.

## Introduction

The hippocampus is important for memory acquisition, storage, recall, and reconstruction [1], [2], [3], it can encode multiplex information associated with specific contexts [4], it is involved in cognitive control [5], and its neural network and cellular physiology are fundamental for these cognitive processes [6], [7]. Hippocampal pyramidal neurons receive a large number of afferent synaptic connections from different brain regions that are potentially capable of inducing long-term synaptic plasticity [8], [9], [10], [11], [12], [13]. Thus, a delicate set of mechanisms is required for these neurons to integrate the information received at different locations and times to decode the external information.

How does the synaptic plasticity-inducing information that reaches a neuron through different synaptic inputs get encoded at the cellular level? We and others proposed a model based on the notion that plastic changes in synapses occur in defined functional dendritic compartments. Synapses expressing synaptic plasticity can interact in specific ways depending on whether they reside within or between functional dendritic compartments [14], [15], [16], [17], [18], [19], [20].

Research on the interaction between LTP and LTD in the rodent hippocampus is abundant [23], [24], [25], [26], [27], [28], [29], [30], [31], [32]. In this study, we further tested the hypothesis of functional compartmentalization by examining the interaction between CA1 synapses expressing long-term synaptic potentiation (LTP) and long-term synaptic depression (LTD), two forms of synaptic plasticity that are considered to be important for the encoding of spatial, contextual and relational information [7], [21], [22].

In the CA1 area, the interaction between LTP and LTD can be interfering [12], [33] or cooperative [19]. To gain insight into how neurons encode spatially and temporally segregated information into their plastic synapses, we characterized the properties of these two types of interactions between LTP and LTD induced 1) at two separate synaptic inputs located within or across morphologically defined CA1 dendritic compartments (basal and apical dendrites), and 2) with different time intervals between inductions. We found that the interaction between LTP and LTD was much stronger within the same dendritic compartment than across basal and apical compartments; supporting the idea of the existence of separate functional compartments in these dendrites [14], [20]. We also found that the nature of the interaction, interference or cooperation, strongly depended on the time interval between inductions. Interference occurred at shorter, while cooperative interactions occurred at longer time intervals. In an attempt to gain insight into the mechanism underlying LTP/LTD interactions, we discovered that intracompartmental interference between LTP and LTD depended on new protein synthesis but not on transcription. We discuss possible cellular mechanisms underlying these interactions and the functional significance of spatial and temporal interactive processes among synapses expressing synaptic plasticity within a neuron for the encoding of multiplex information.

## Results

**Figure 1:**
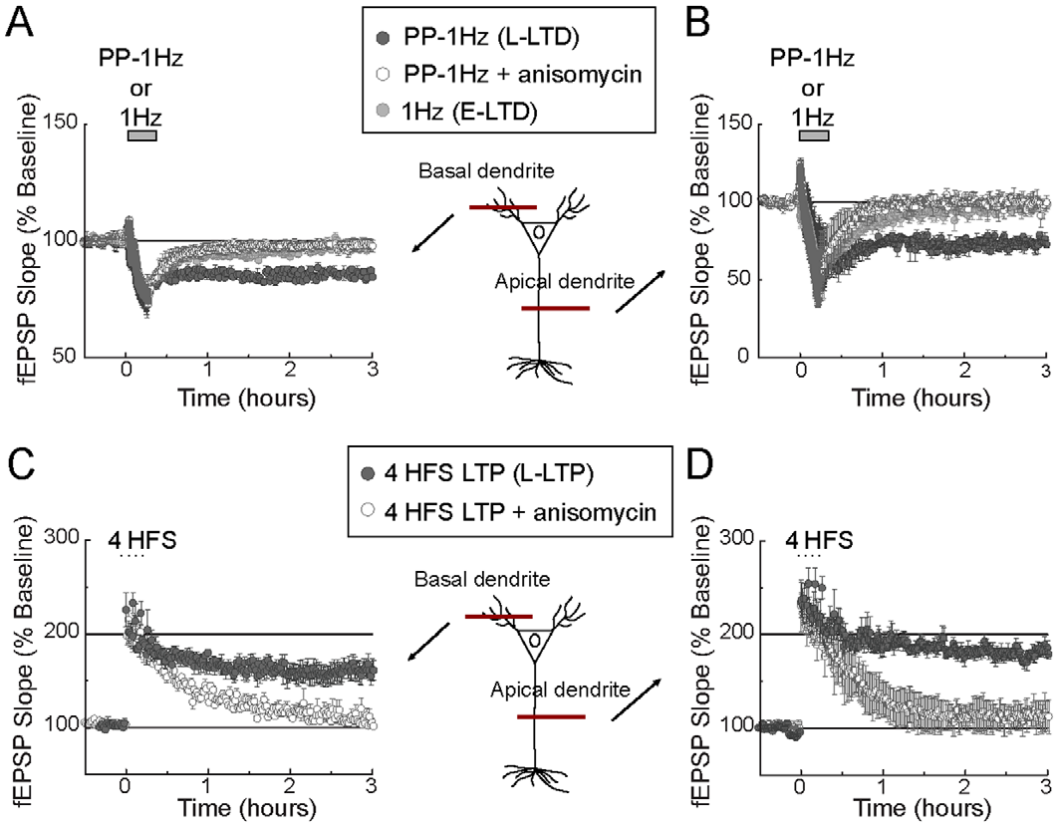
Expression of strong and weak forms of LTP and LTD in the basal and apical dendritic compartments of CA1 pyramidal neurons. (**A, B**): A strong form of LTD that expressed the protein synthesis dependent late phase of LTD (L-LTD) was induced by paired pulses of low frequency stimulation (PP-1Hz: 50 msec interpulse interval at 1 Hz for 15 min) in the basal (**A, dark gray circles**, n = 6) and apical (**B, dark gray circles**, n = 6) dendritic compartments. Conversely, a weak form of LTD that expressed the protein synthesis independent early phase of LTD (E-LTD) was induced after a single train of low frequency stimulation (1 Hz: 1 train of 15 min at 1 Hz) in the basal (**A, light gray circles**, n = 5) and apical (**A, light gray circles**, n = 5) dendritic compartments. Blockage of new protein synthesis transformed strong LTD into weak LTD in the basal (**A, open circles**, n = 6) and apical (**B, open circles**, n = 6) dendritic compartments (anisomycin, an inhibitor of translation, was added between −20 min and +20 min of recording). (**C, D**): A strong form of LTP that expressed the late phase of LTP (L-LTP) was induced by 4 trains of high frequency stimulation (HFS, 4 trains of 1-sec at 100 Hz stimulation, 5 min inter-train interval) in the basal (**C, dark gray circles**, n = 6) and apical (**D, dark gray circles**, n = 6) dendritic compartments. Like with strong LTD, blockage of new protein synthesis transformed strong LTP into weak LTP that expressed the early phase of LTP in the basal (**C, open circles**, n = 6) and apical (**D, open circles**, n = 6) dendritic compartments (anisomycin was added between −20 min and +20 min of recording). S1 and S2 represent independent afferents synapsing on basal or apical dendrites, respectively.

LTP and LTD are not unitary phenomena and can be induced in synapses by a number of stimulation protocols [21]. In the choosing of the stimulation protocols to induce LTP and LTD, we considered a central element of their expressions: the requirement of new protein synthesis. A protein synthesis dependent form of LTD expressing the late phase of LTD (strong LTD) can be induced by delivering one train of paired-pulse low frequency stimulation (PP-1 Hz, 1 train of 50-msec paired pulses at 1 Hz for 15 min) to afferent axons synapsing on the basal (Fig. 1A, Table 1) or the apical (Fig. 1B, Table 1) dendritic compartments of CA1 pyramidal neurons of the mouse hippocampus. A protein synthesis dependent form of LTP expressing the late phase of LTP (strong LTP) can be induced by delivering four repetitive trains of high frequency stimulation (4 HFS, four trains of 1-sec stimulation at 100 Hz, 5 min inter train interval) to afferent axons synapsing on the basal (Fig. 1C) or the apical (Fig. 1D) dendritic compartments of CA1 pyramidal neurons of the mouse hippocampus. We used these induction protocols to characterize the interference between strong LTP and strong LTD.

**Table 1:** Dependency on transcription and protein synthesis inhibitors of the expression of LTD in the basal and apical dendritic compartments.

	Basal	LTD	Apical	LTD
Time period	30–60 min	120–150 min	30–60 min	120–150 min
*CONTROL*	*85±3%*	*85±6%*	*69±4%*	*73±6%*
Actinomycin-D	89±3%	92±5%	79±6%	**95±5%** *
Anisomycin	**94±4%** *	**98±3%** *	**91±5%** *	**99±4%** *

The values represent the relative change in fEPSP amplitude with respect to the baseline (100%).

*Statistically significant from control at p<0.05.

A protein synthesis independent form of LTD expressing the early phase of LTD (weak LTD), can be induced by delivering one train of low frequency of stimulation (1 Hz, 900 pulses at 1 Hz for 15 min) to afferent axons synapsing on the basal (Fig. 1A) or the apical (Fig. 1B) dendritic compartments of CA1 pyramidal neurons of the mouse hippocampus. This stimulation protocol and the one for strong LTP were used to characterize the cooperative interaction between LTP and LTD (strong LTP + weak LTD).

### A potent interference is observed between strong forms of LTP and LTD within the same dendritic compartment

In an earlier report, we found that the interaction between a weak and strong form of LTP is compartmentalized within morphologically defined dendritic domains [14]. Here we ask whether the interaction between strong forms of LTP and LTD is compartmentalized too.

**Figure 2:**
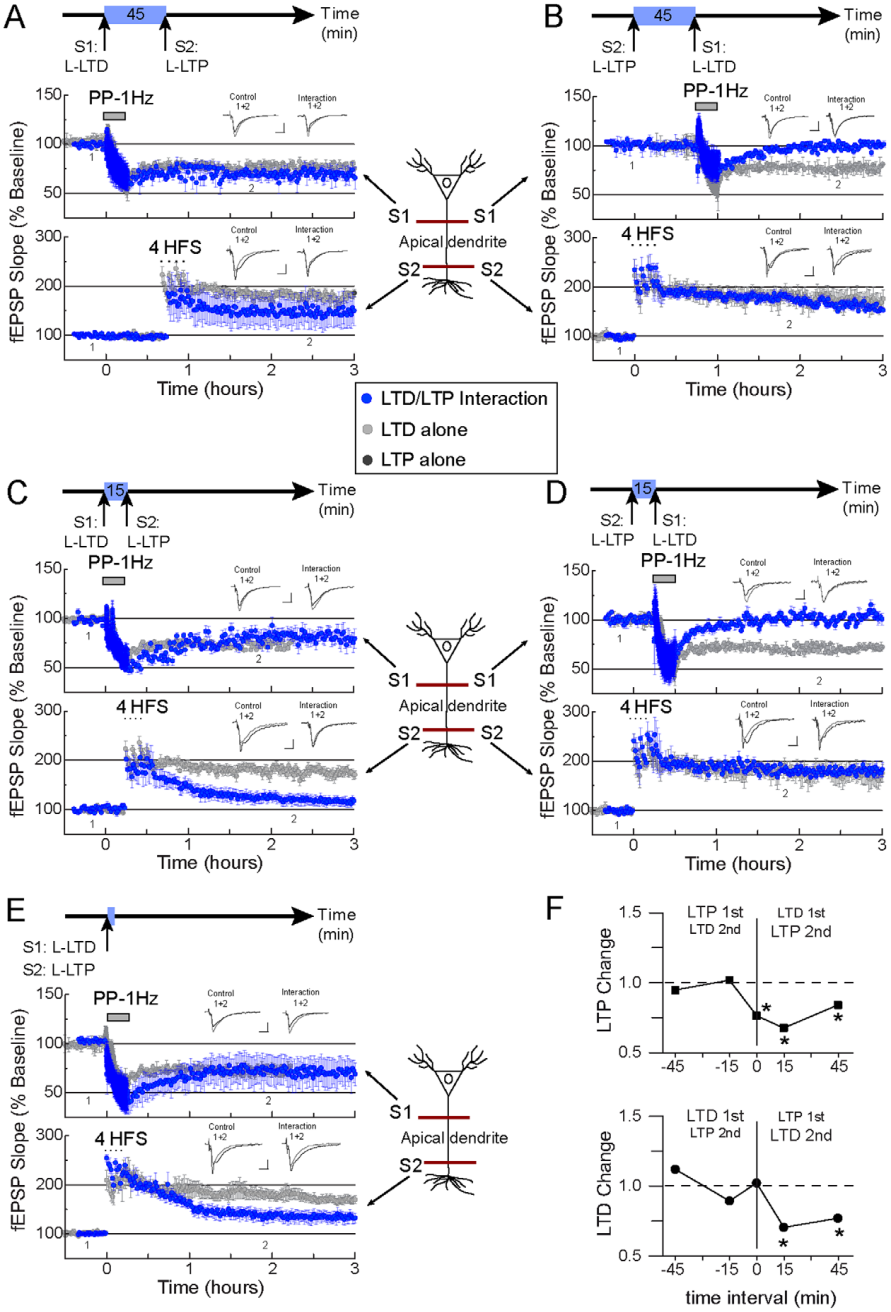
Severe interference between strong forms of LTP and LTD within the same dendritic compartment. (**A**): Strong LTD induced in the apical pathway S1 remains unaltered (top blue trace), while the subsequent expression of strong LTP induced in the apical pathway S2 is reduced (bottom blue trace). Time interval between inductions is 45 min. To facilitate visualization of the interference, the expression of control (unpaired) strong LTD (top) and strong LTP (bottom) is shown in all panels (grey traces). (**B**): Reversing the roles produces an even stronger interference. Strong LTP induced in the apical pathway S2 (bottom blue trace) blocks the subsequent expression of strong LTD induced in the apical pathway S1 (top blue trace). Time interval between inductions is 45 min. (**C**): The interference of strong LTD (apical pathway S1, top blue trace) over the expression of strong LTP (apical pathway S2, bottom blue trace) is much more substantial with a shorter time interval between inductions (15 min). (**D**): With the same time interval (15 min), strong LTP (apical pathway S2, bottom blue trace) blocks the subsequent expression of strong LTD (apical pathway S1, top blue trace). (**E**): Despite the apparent dominance of the interference of LTP over LTD, simultaneous induction of strong LTD (apical pathway S1, top blue trace) and strong LTP (apical pathway S2, bottom blue trace) results in reduced expression of LTP. (**F**): Graphs representing LTP change (top) and LTD change (bottom) indexes (see text for details). Negative time intervals correspond to the change in the first induced form of synaptic plasticity, positive time intervals correspond to the change in the second (subsequent) form of synaptic plasticity. Note that either LTP or LTD when first induced shows an index change around 1 (no interference). In contrast, LTP and LTD change indexes are smaller than 1 (interference) for the second induced form of plasticity. Also note that as the time interval between inductions increases the magnitude of interference (measured as the LTP or LTD change) decreases for the second induced form of plasticity. At time interval 0 min, LTP change is smaller than 1, while LTD change is about 1. Each independent data set was obtained from 6 mice. S1 and S2 represent independent afferents synapsing on the apical dendritic compartment. Representative traces are shown (gray: control; dark gray: interaction; 1: baseline, 2 after synaptic plasticity induction). Scale bar is 2 mV and 5 msec. Enlarged traces are shown in Fig. S1.

We first explored a potential interaction between strong forms of LTP and LTD by inducing each type of synaptic plasticity in two separate (and independent, see Methods) pathways synapsing within the same dendritic compartment (apical dendrites within the stratum radiatum). When separated by a 45 min time interval, induction of the strong form of LTD (Fig. 2A S1, Table 2, Table S1, Enlarged traces are shown in Fig. S1) significantly impaired the subsequent expression of LTP (Fig. 2A S2, Table 2, Table S1). When we inverted the order of induction (LTP and then LTD), we observed that induction of the strong form of LTP (Fig. 2B S2, Table 2, Table S1) impaired the subsequent expression of LTD (Fig. 2B S1, Table 2, Table S1). With a 15 min time interval between strong LTP and strong LTD inductions, we observed that LTD occluded the subsequent expression of LTP (Fig. 2C, Table 2, Table S1); and when the sequence of induction was reversed (LTP and then LTD), we observed that LTP occluded the subsequent expression of LTD (Fig. 2D, Table 2, Table S1). When LTP and LTD were both induced at the same time, we observed that LTD induction occluded LTP expression (Fig. 2E, Table 2; Table S1).

**Table 2:** Intracompartmental interaction between strong forms of LTP and LTD in the apical dendritic compartment.

	LTP 120–150 min	LTD 120–150 min
*CONTROL*	*189±6%*	*74±4%*
LTD before LTP 45′	**147±6%** *	69±4%
LTD before LTP 15′	**118±4%** *	82±4%
LTD & LTP	**136±3%** *	71±3%
LTP before LTD 15′	178±6%	**102±2%** *
LTP before LTD 45′	168±5%	**99±2%** *

The values represent the relative change in fEPSP amplitude with respect to the baseline (100%).

*Statistically significant from control at p<0.05.

In order to quantify the degree of interaction between LTP and LTD, we calculated an index factor which represents the amplitude change in the expression of either form of synaptic plasticity during the interaction, compared to their corresponding controls (Fig. 2F; see Methods for details). For instance, an LTP or LTD change (or index factor) equal to 1, represents that the expression of LTP or LTD was not affected during the interaction. An LTP or LTD change smaller than 1 (index change <1), represents that LTP or LTD expression was negatively or antagonistically affected during the interaction; and in contrast, an LTP or LTD change larger than 1 (index change >1), represents that LTP or LTD expression was positively or cooperatively affected during the interaction. From this analysis (shown in Fig. 2F), we can extract three main conclusions: 1) That during sequential induction of strong forms of LTP and LTD, the subsequent form of synaptic plasticity was always strongly affected, while the initial remained minimally changed. This becomes apparent by looking at the index change of the first induction compared to the index change of the second induction (Fig. 2F, first induction is plotted with negative time intervals and second induction is plotted with positive intervals). While the LTP and LTD change was close to 1 when first induced, the LTP and LTD change diverged from 1 (0.75 averaged value) when induced second. 2) That the magnitude of interaction decreases in relation to the time interval between inductions. The index analysis reveled that the expression of the second form of synaptic plasticity was more affected with the 15 min time interval (Fig. 2F, LTP change = 0.68, LTD change = 0.71; each index value different from 1, p<0.05) than with the 45 min time interval (LTP change = 0.84, LTD change = 0.77; each index value different from 1, p<0.05). 3) That when simultaneously induced, induction of strong LTD overpowers LTP expression (Fig. 2F, LTD change = 1.02, index value not different from 1, p>0.05; LTP change = 0.76; index value different from 1, p<0.05).

### Only a modest interference is observed between strong forms of LTP and LTD across separate dendritic compartments

**Figure 3:**
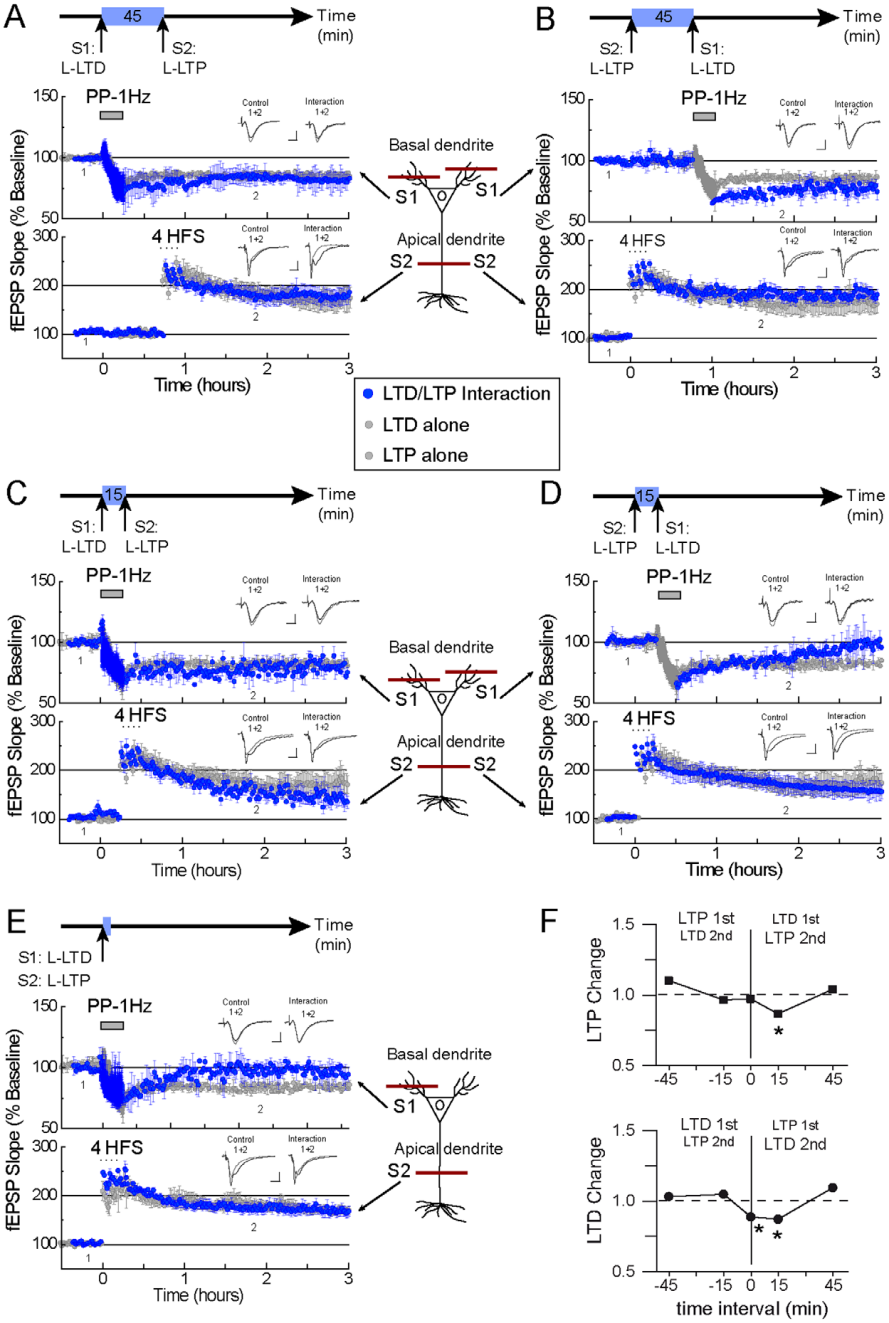
Mild interference between strong forms of LTP and LTD across dendritic compartments. (**A**): Absence of interference between strong LTD induced in the basal dendritic compartment (pathway S1, top blue trace) and strong LTP induced in the apical dendritic compartment (pathway S2, bottom blue trace). Time interval between inductions is 45 min. To facilitate visualization of interference, the expression of control (unpaired) strong LTD (top) and strong LTP (bottom) is shown in all panels (grey traces). (**B**): Similarly, strong LTP induced in the apical pathway S2 (bottom blue trace) does not interfere with the subsequent expression of strong LTD induced in the basal pathway S1 (top blue trace). Time interval between inductions is 45 min. (**C**): A mild interference of strong LTD (basal pathway S1, top blue trace) over the expression of strong LTP (apical pathway S2, bottom blue trace) is observed with a 15 min time interval between inductions. (**D**): A modest interference is also observed for strong LTP (apical pathway S2, bottom blue trace) over the expression of strong LTD (basal pathway S1, top blue trace) with a 15 min time interval. (**E**): In spite of the observed mild transcompartmental interference, simultaneous induction of strong LTD (basal pathway S1, top blue trace) and strong LTP (apical pathway S1, bottom blue trace) results in blockage of the expression of strong LTD. (**F**): Graphs representing LTP change (top) and LTD change (bottom) indexes (see text for details). Negative time intervals correspond to the change in the first induced form of synaptic plasticity, positive time intervals correspond to the change in the second (subsequent) form of synaptic plasticity. Similar to the intracompartmental studies, when first induced, LTP and LTD change indexes show values close to 1 (no interference). LTP and LTD change indexes are smaller than 1 (interference) for the second induced form of plasticity only at 15 min. time interval, as no interaction was observed at 45 min. time interval (LTP and LTD change index∼1). At time interval 0 min, LTD change is smaller than 1, while LTP change is about 1. Each independent data set was obtained from 6 mice. S1 and S2 represent independent afferents synapsing on the basal and the apical dendritic compartments. Representative traces are shown (gray: control; dark gray: interaction; 1: baseline and 2: after synaptic plasticity induction). Scale bar is 2 mV and 5 msec. Enlarged traces are shown in Fig. S2.

We next studied the interaction between strong LTP and LTD across dendritic compartments. When separated by a 45 min time interval, induction of the strong form of LTD (Fig. 3A S1, Table 3, Table S2, Enlarged traces are shown in Fig. S2) did not impair the subsequent expression of LTP (Fig. 3A S2, Table 3; Table S2). When we inverted the order of induction (LTP and then LTD), we also did not observe that induction of the strong form of LTP (Fig. 3B S2, Table 3, Table S2) impaired the subsequent expression of LTD (Fig. 3B S1, Table 3, Table S2). Quantitative analysis revealed indexes of 1.04 and 1.09 for the second LTP and LTD expressions, respectively (Fig. 3F; each index value not different from 1, p>0.05). With a 15 min time interval between inductions, we observed that LTD induction significantly decreased the subsequent expression of LTP (Fig. 3C, Table 3, Table S2); and when the sequence of induction was reversed (LTP and then LTD), we observed that LTP also significantly decreased the subsequent expression of LTD (Fig. 3D, Table 3, Table S2). Similar to what was observed for the interaction within the same dendritic compartment, we observed here that the first form of synaptic plasticity affected the expression of the subsequent one, irrespective of the nature of it (Fig. 3F; 1^st^: LTP change = 0.99, LTD change = 1.02; each index value not different from 1, p>0.05; 2^nd^: LTP change = 0.87 and LTD change = 0.87; each index value different from 1, p<0.05). In contrast to what was observed for the interaction within the same dendritic compartment, when LTP and LTD were both induced simultaneously across separate dendritic compartments, we observed that LTP induction occluded LTD expression (Fig. 3E, Table 3, Table S2). The change indexes were 0.97 for LTP (index value not different from 1, p>0.05) and 0.89 for LTD (index value different from 1, p<0.05) (Fig. 3F).

**Table 3:** Transcompartmental interaction between strong forms of LTP and LTD (LTP and LTD are induced at apical and basal dendrites, respectively).

	LTP apical 120–150 min	LTD basal 120–150 min
*CONTROL*	*182±9%*	*82±6%*
LTD before LTP 45′	176±9.5%	81±3%
LTD before LTP 15′	**146±8%** *	79±7%
LTD & LTP	173±9%	**97±4.5** *
LTP before LTD 15′	164±5%	**95±5.5%** *
LTP before LTD 45′	188±11%	78±3%

The values represent the relative change in fEPSP amplitude with respect to the baseline (100%).

*Statistically significant from control at p<0.05.

Our data suggest that the interaction between strong forms of LTP and LTD is largely, but not completely, compartmentalized. Significant interference was observed for intracompartmental LTP and LTD interactions at all tested time intervals (0–45 min; Table 2; Fig. 2F). Transcompartmental interference, however, occurred only when the time interval between LTP and LTD inductions was 15 min or 0 min, but not at 45 min (Table 3, 4 and Fig. 3F). A temporal dependency was not the only difference between intra and transcompartmental interactions, the magnitude of the transcompartmental interference was smaller than the intracompartmental interference (Table 2, 3, 4, and compare the magnitude of LTP and LTD changes in Fig. 2F and 3F). With regard to the dominance of one form of synaptic plasticity over the other, we found that although LTP seems to be dominant over LTD (Table 2 and 3), our data indicate that the sequence of induction is more important. Typically, the prior form of plasticity overpowered the subsequent one (Fig. 2F and Fig.3F). Interestingly, when we examined the interaction between LTP and LTD induced simultaneously, we found that whereas LTD overpowered LTP within the same dendritic compartment (Fig. 2E, Table 2), LTP overpowered LTD across dendritic compartments (Fig. 3E, Tables 3 & 4).

**Table 4:** Transcompartmental interaction between strong forms of LTP and LTD (LTP and LTD are induced at basal and apical dendrites, respectively).

	LTP basal 120–150 min	LTD apical 120–150 min
*CONTROL*	*159±7%*	*70±5%*
LTD before LTP 45′	158±10%	75±7%
LTD before LTP 15′	**120±9%** *	77±5%
LTD & LTP	150±10%	**93±5%** *
LTP before LTD 15′	162±10%	**97±6%** *
LTP before LTD 45′	167±10%	82±5%

The values represent the relative change in fEPSP amplitude with respect to the baseline (100%).

*Statistically significant from control at p<0.05.

### The cooperative interaction between a weak and a strong form of LTP and LTD is both compartmentally restricted and temporally constrained

**Figure 4:**
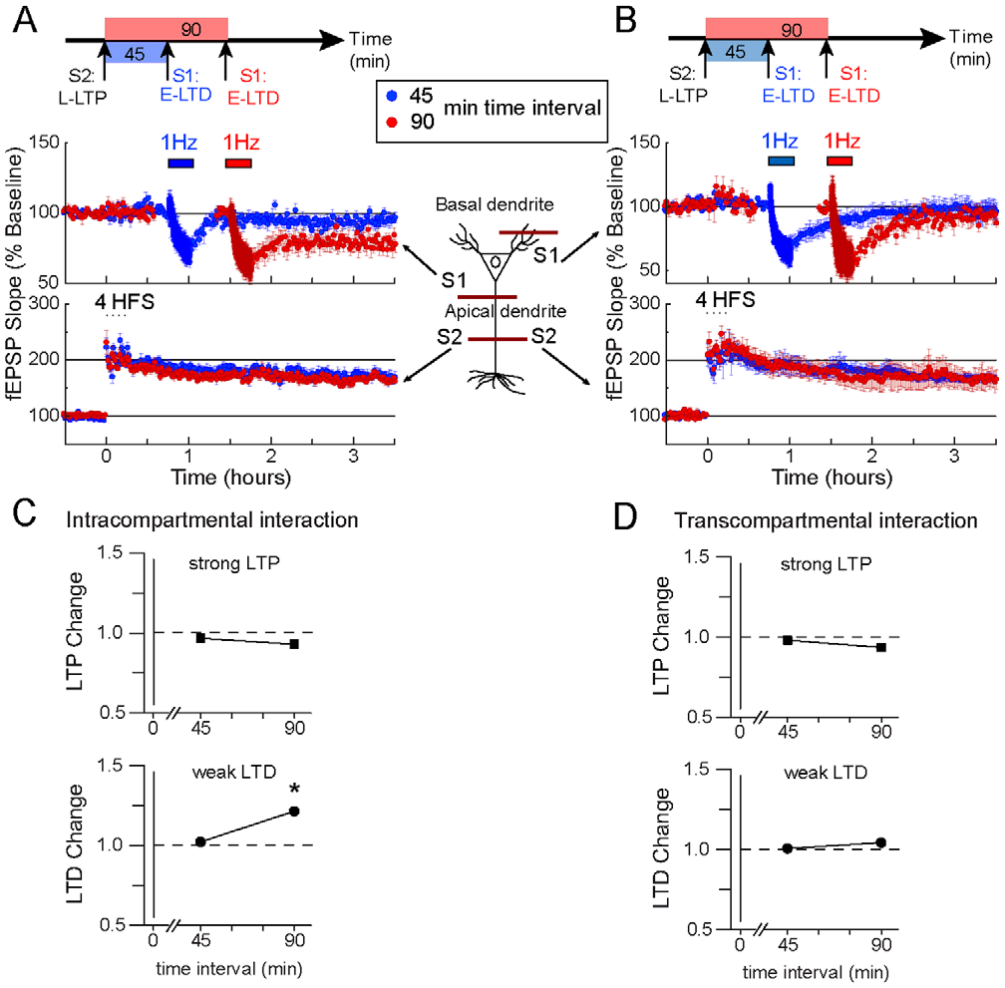
Temporal restriction and dendritic compartment specificity for the cooperative interaction between LTP and LTD. (**A**): Failure to induce a cooperative interaction (the conversion of weak LTD into strong LTD) between strong LTP induced in apical pathway S2 (bottom blue trace) and weak LTD induced in apical pathway S1 (top blue trace) with a 45 min time interval between inductions. However with a 90 min time interval between inductions (red traces), cooperative interaction is observed. (**B**): Absence of cooperative interaction between strong LTP and weak LTD across dendritic compartments. Weak LTD induced in the S1 basal pathway (top panel) is not transformed into strong LTD after induction of strong LTP in the apical pathway S1 (bottom panel) with either, a 45 min (blue traces) or a 90 min (red traces) time interval between inductions. (**C**): Graphs representing the LTP change (top) and LTD change (bottom) indexes for the intracompartmental interaction between a strong form of LTP and a weak form of LTD. An absence of a cooperative effect is evidenced by LTP and LTD change indexes close to 1. In contrast, an LTD change index higher than 1 demonstrated a cooperative effect between LTP and LTD with a time interval of 90 min. The LTP change index remained close to 1. (**D**): Graphs representing the LTP change (top) and LTD change (bottom) indexes for the transcompartmental interaction between a strong form of LTP and a weak form of LTD. No deviation from 1 is observed for the LTP or LTD change index at 45 or 90 min time interval; demonstrating the absence of cooperative interaction between LTP and LTD across dendritic compartments. In all the figures each independent data set was obtained from 6 mice. S1 and S2 represent two independent afferents synapsing on the apical (A) or on the basal and the apical dendritic compartment, respectively (B).

We have characterized the interaction between strong forms of LTP and LTD within and across dendritic compartments and found that it is antagonistic and largely compartmentalized. A study from Sajikumar and Frey reported a cooperative interaction between LTP and LTD [19]. In their study, weak and strong forms of LTD and LTP were induced within the same apical dendritic compartment. This pairing produced enhancement of the weak form of synaptic plasticity (either LTP or LTD). Here, we examined whether the cooperative interaction between the weak form of LTD and the strong form of LTP is compartmentally restricted. Unexpectedly, we did not observe a cooperative interaction between weak LTD and strong LTP with a 45 min time interval between inductions (Fig. 4A, blue trace, S1, Fig. 4C: LTD change = 1.02; index value not different from 1, p>0.05). Failure to observe the cooperative interaction with a 45 min time interval also occurred when pairing strong and weak forms of LTD and LTP, respectively (not shown). Only when we lengthened the time interval between inductions to 90 min did we observe a cooperative interaction (Fig. 4A S1; Fig. 4C: LTD change = 1.21; index value different from 1, p<0.05). It is important to notice that the proposed mechanism, based on the Synaptic Tagging and Capture Model (STC), for the cooperative interaction poses that new plasticity products (protein and mRNA) generated by the induction of the strong form of synaptic plasticity can also be productively used by the weak form of synaptic plasticity; a process sure to occur within a range of 45 min between the induction of strong and weak forms of synaptic plasticity [18], [19].

Next, we tested whether the cooperative interaction between LTP and LTD could also occur across dendritic compartments. Preceded by the induction of a strong form of LTP in the apical dendritic compartment, induction of weak LTD in the basal dendritic compartment did not result in the expression of an enduring (strong) form of LTD. The transcompartmental cooperative interaction failed with a 45 or 90 min time interval between inductions (Fig. 4B and Fig. 4D: LTD change at 45 min = 1.01, at 90 min = 1.04; each index value not different from 1, p>0.05), or with a 15 or 120 min time interval (not shown). Similarly, we observed no interaction when a strong form of LTD was induced prior to a weak form of LTP (not shown).

Our data suggest that the cooperative interaction between weak and strong forms of LTP and LTD is restricted within the same dendritic compartment, and it only occurs with a 90 min, but not a 45 min, time interval between LTP and LTD inductions.

### The expression of the interference between LTP and LTD is also temporally constrained

**Figure 5:**
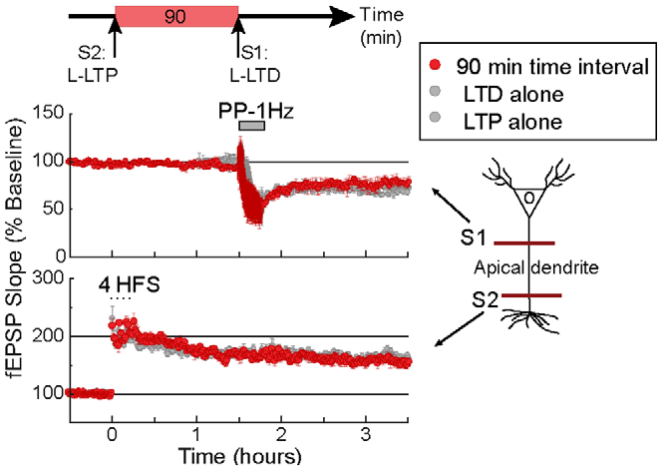
A critical period for the expression of the interference between LTP and LTD. Absence of intracompartmental interference between strong LTP (apical pathway S2, bottom red trace) and strong LTD (apical pathway S1; top red trace) with a 90 min time interval between inductions. The expression of control (unpaired) strong LTD (top) and strong LTP (bottom) is shown in the gray traces. Compare with the intracompartmental interference observed with a 45 min time interval between inductions (Fig. 2B). Also compare the interference observed with a 15 min time interval and with a 45 min time interval between inductions across dendritic compartments (e.g. Fig. 3D vs. Fig. 3A). Each independent data set was obtained from 6 mice. S1 and S2 represent independent afferents synapsing on the apical dendritic compartment.

In addition to the compartmentally restricted nature of the interactions between LTP and LTD, there appears to be a temporal restriction that regulates the interfering and cooperative nature of the LTP/LTD interactions. Interference between LTP and LTD across separate dendritic compartments is observed with 0 and 15 min, but not with a 45 min time interval between inductions (Fig. 3F). Interference between LTP and LTD within the same dendritic compartment is observed with 0, 15 and 45 min time intervals between inductions (Fig. 2F). Hence, we speculate that if a longer time interval between LTP and LTD inductions is used, intracompartmental interference would also disappear -just as we see for transcompartmental interference with a 45 min time interval. Therefore, we tested the intracompartmental interaction between strong forms of LTP and LTD with a time interval between inductions of 90 min. As predicted, we found no interference between LTP and LTD with this time interval between inductions (Fig. 5: LTD change at 90 min = 0.92; index value not different from 1, p>0.05).

These data support the idea that the interference between strong forms of LTP and LTD is limited to a certain time window between LTP and LTD inductions. Importantly, in Fig. 4 we showed that cooperative interaction between LTP and LTD within the same dendritic compartment was observed with 90 but not 45 min time interval between inductions (Fig. 4A). Here, we show that the interference between LTP and LTD was no longer observed with a 90 min time interval between inductions (see Table 5). A natural speculation from these observations is that concomitant with the failure to express interference is the ability to successfully express cooperative interactions. Hence, it is possible that these two phenomena are mechanistically related (see Discussion for proposed model).

**Table 5:** Interactions between LTP and LTD within the same apical (INTRA) and across dendritic (TRANS) compartments.

	TI	0 min	15 min	45 min	90 min
	FP				
**INTRA**	Weak + Strong	nd	*No interaction*	*No interaction*	Cooperation
			*Index≈1*	*Index≈1*	**Index>1**
	Strong + Strong	Interference	Interference	Interference	*No interaction*
		**Index<1**	**Index<1**	**Index<1**	*Index≈1*
**TRANS**	Weak + Strong	nd	*No interaction*	*No interaction*	*No interaction*
			*Index≈1*	*Index≈1*	*Index≈1*
	Strong + Strong	Interference	Interference	***No interaction***	nd
		**Index<1**	**Index<1**	*Index≈1*	

**TI**: Time interval. **FP**: Form of synaptic plasticity. *No Interaction* indicates that we observed neither cooperation nor interference between LTP and LTD. nd: not determined. The index is the observed change in synaptic plasticity as in Fig. 7A.

### The intracompartmental interference between LTP and LTD depends on protein synthesis

**Figure 6:**
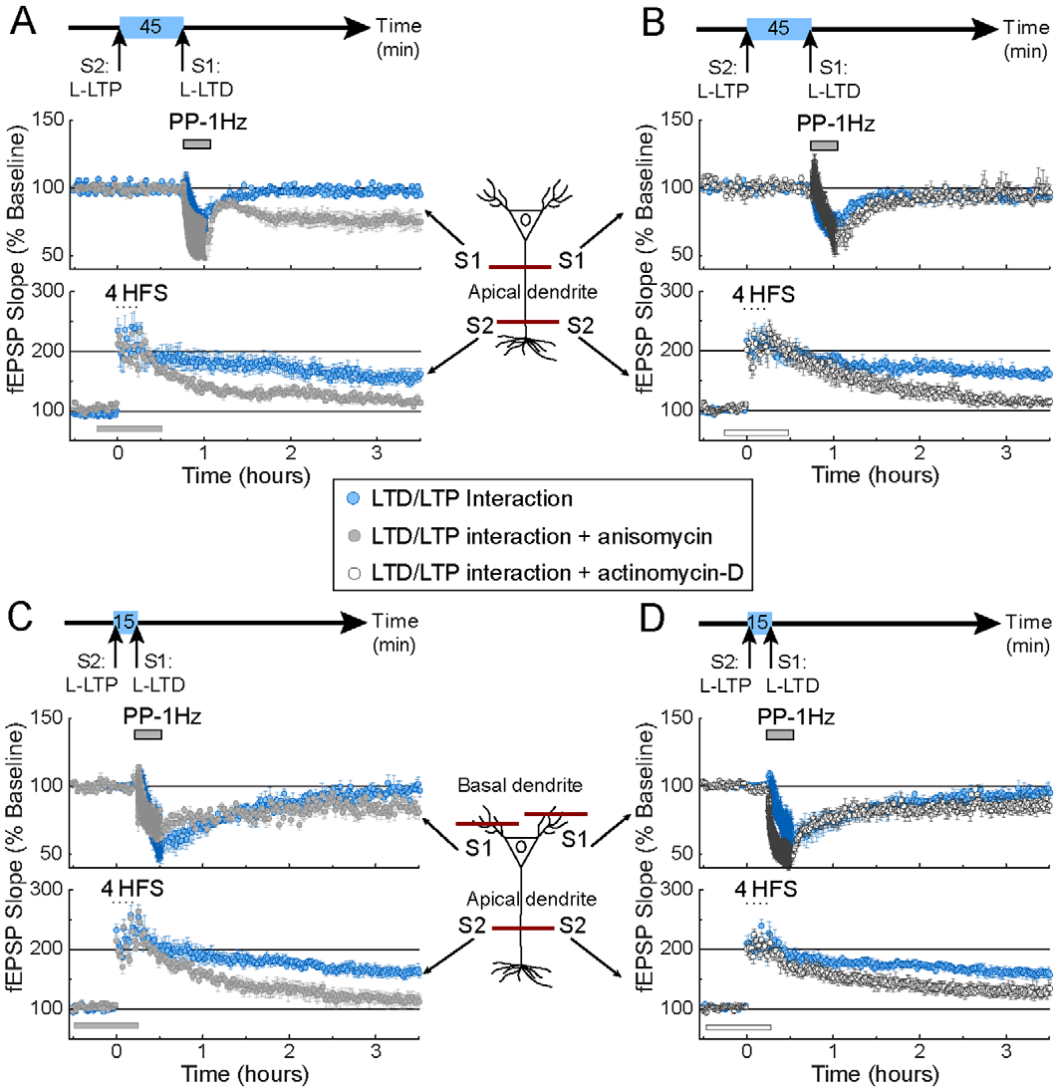
Dependency on protein synthesis and transcription of the interference between LTP and LTD. Within the same dendritic compartment: (**A**): Disruption of the expression of strong LTP (apical pathway S2, bottom gray trace) by the protein synthesis blocker anisomycin (20 µM, horizontal bar) prevents the interference over the subsequent expression of strong LTD (apical pathway S1, top gray trace). Time interval between inductions is 45 min. To facilitate visualization of the rescue of the interference, the expression of the interaction between strong LTP and strong LTD in normal *r*ACSF (without blockers) is shown in light blue traces in all panels. (**B**): Disruption of the expression of strong LTP (apical pathway S2, bottom hollow trace) by the transcription blocker actinomycin (40 µM, horizontal bar) did not prevent the interference over the subsequent expression of strong LTD (apical pathway S1, top hollow trace). Time interval between inductions is 45 min. *Across dendritic compartments*. (**C**): Disruption of the expression of strong LTP (apical pathway S2, bottom gray trace) by anisomycin (horizontal bar) did not significantly prevent the interference over the subsequent expression of strong LTD (basal pathway S1, top gray trace). Time interval between inductions is 15 min. (**D**): Disruption of the expression of strong LTP (apical pathway S2, bottom hollow trace) by actinomycin (horizontal bar) did not significantly prevent the interference over the subsequent expression of strong LTD (basal pathway S1, top hollow trace). Time interval between inductions is 15 min. In all the figures each independent data set was obtained from 6 mice. S1 and S2 represent two independent afferents synapsing on the apical (A, C) or on the basal and the apical dendritic compartment, respectively (B, D).

What is the molecular basis of the interference between LTP and LTD? In an early study, Muller et al (1995) demonstrated that competition between LTP and LTD depended on NMDA receptor and calcineurin activation; pointing out the crucial role of induction mechanisms for the expression of the interfering effect between LTP and LTD [33]. Here, we studied the interaction between strong -and protein synthesis dependent- forms of LTP and LTD. Thus, we investigated whether *de novo* translation, a fundamental cellular process for the expression of the late phase of synaptic plasticity [7], underlies the interference between LTP and LTD. We also examined the role of transcription on the expression of the intra and transcompartmental interference between strong forms of LTP and LTD. To test the effect of translation or transcription blockage on the LTP and LTD interference, we chose the 45 min time interval between LTP and LTD for intracompartmental interference, and the 15 min time interval for transcompartmental interference. Blockage of the late expression of LTP with the protein synthesis inhibitor anisomycin did rescue the interfering effect of LTP over the subsequent expression of LTD (Fig. 6A, Vehicle: LTP_[120–150 min]_ = 166±10%, LTD_[120–150 min]_ = 98±4%; Anisomycin: LTP_[120–150 min]_ = 129±7%, LTD_[120–150 min]_ = 77±4%; p<0.05 for vehicle versus anisomycin LTP and LTD pairs). In contrast, disruption of the late expression of LTP with the transcription inhibitor actinomycin-D did not rescue the interfering effect of LTP over the subsequent expression of LTD (Fig. 6B, Vehicle: LTP_[120–150 min]_ = 171±5%, LTD_[120–150 min]_ = 93±7%; Actinomycin-D: LTP_[120–150 min]_ = 130±15%; LTD_[120–150 min]_ = 94±6%; p<0.05 for LTP pairs and p>0.05 for LTD pairs).

We next investigated the dependency on protein synthesis and transcription of the interference between LTP and LTD across dendritic compartments. Disruption of the late expression of LTP by anisomycin in the apical dendritic compartment did not rescue the interfering effect over LTD induced in the basal dendritic compartment (Fig. 6C, Vehicle, LTP _[120–150 min]_ = 173±9%, LTD_[120–150 min]_ = 90±2%; Anisomycin: LTP_[120–150 min]_ = 128±6%, LTD_[120–150 min]_ = 84±9%; p<0.05 for LTP pairs and p>0.05 for LTD pairs). Similarly, blockage of the late expression of LTP by actinomycin in the apical dendritic compartment did not prevent the interfering effect over the expression of the basal LTD (Fig. 6D, Vehicle: LTP_[120–150 min]_ = 170±10%, LTD_[120–150 min]_ = 89±2%; Actinomycin-D: LTP_[120–150 min]_ = 139±22%; LTD_[120–150 min]_ = 82±12%; p<0.05 for LTP pairs and p>0.05 for LTD pairs). We cannot, however, rule out with certainty that failure to observe transcompartmental rescue is not due to a lingering effect of each drug after wash out (however see Methods).

These findings uncover a requirement for new protein synthesis, but not transcription, of the intracompartmental interference between LTP and LTD, whereas each process seems to not have substantial role in the transcompartmental interference.

## Discussion

What are the cellular mechanisms that dictate and coordinate the interactions between synapses allowing information to be associated, segregated, and dismissed as appropriate for a given experience? Consider a neuron with synapses being activated by different learning-associated information streams. During initial experience such activation elicits synaptic plasticity at a subset of the synapses, a phenomenon thought to be the cellular substrate of memory [3], [34]. While learning prompts the storage of different bits of information in multiple synapses of the cell by means of synaptic plasticity, the interactions between these plastic synapses, a process that we call “*synaptic plasticity interactions*”, are hypothesized to be key elements for the association and segregation of synaptic plasticity-encoded information induced in different synapses of the same cell [14], [20], [35], [36]. We speculate that induction of synaptic plasticity can form particular domains of plasticity-associated metabolic activity within dendrites; a process that we called “functional compartmentalization”. Thus, induction of synaptic plasticity may form functional groups of plastic synapses within morphologically defined dendritic compartments. And it is within and between these “functional compartments” that the proper association and segregation of learning-induced plastic changes takes place.

Neurons in the hippocampus receive inputs from different brain areas that need to be integrated for proper encoding of information [8], [9], [10], [11], [13]. The encoding properties of a hippocampal neuron may conceivably comprise the integration of multiple forms of synaptic plasticity elicited at separate synapses of the same neuron [14], [15], [18], [19], [20], [36], [37].

In this study we investigated the interaction between LTP and LTD elicited in separated synaptic inputs to a CA1 pyramidal neuron of the mouse hippocampus and found that it is temporally and spatially regulated. In particular, there appear to be functionally separate dendritic compartments corresponding to the anatomical domains we used for inducing LTP and LTD. Our findings further highlight the integrative capability of CA1 neurons of the mouse hippocampus, particularly, in regard to the induction and expression of opposing forms of synaptic plasticity within the same cell [12], [25], [38], [39], [40], [41], [42], [43]. Our main findings are summarized as follows: 1) that intracompartmental interactions are stronger in magnitude than transcompartmental ones, 2) that the magnitude of the interaction depends on the time separation between LTP and LTD inductions, 3) that during intracompartmental interference between LTP and LTD, only the subsequent form of synaptic plasticity is affected, 4) that cooperation and interference between LTP and LTD can not occur at the same time intervals, and 5) that the intracompartmental interference between LTP and LTD depends on new protein synthesis.

### Intracompartmental protein synthesis dependent LTP/LTD interference

Our finding that the interference between LTP and LTD depends on the synthesis of new proteins suggests that proteins generated in response to either strong LTP or strong LTD –inducing stimuli might restrict the expression of an opposite form of synaptic plasticity. We suggest that cooperative interactions could only take place when this interfering activity is reduced. Thus, in addition to previously described mechanisms contributing to the interference between LTP and LTD [12], [33], our study shows *de novo* synthesis of protein as a novel mechanism for this interference. The requirement of protein synthesis would represent a mechanistic switch that implies a higher activity threshold for a long lasting interference whose functional compartmentalization could rely on mechanisms for homeostatic regulation [46]. The absence of dependency on transcription blockage for the LTP/LTD interference supports a specific role of protein synthesis on this phenomenon.

What could be the signaling pathways underlying the protein synthesis dependent interference? Two possible candidate pathways emerge, the protein kinase A (PKA) pathway for LTP and the metabotropic glutamate receptor (mGluR) pathway for LTD. Each of these signaling pathways is known to elicit the protein synthesis dependent phase of the strong forms of LTP and LTD used in this study, respectively [47], [48], [49], [50].

If synthesis of protein factors is required for the LTP/LTD interference, where does this protein synthesis occur? The location could be somatic, synaptic or both. Somatic protein synthesis would allow the seeding of protein along the somatodendritic axis. Somatic protein synthesis could follow nuclear activation by the signal generated at the stimulated synapses in response to strong synaptic plasticity induction that travels back to the nucleus and triggers gene transcription [7]. This scenario would require target-directed trafficking of new proteins towards the active (functional) dendritic compartment [51], [52] (conjectured in [47]). Synaptic protein synthesis [53], [54], [55], [56] appears less likely because it would require synaptically produced proteins to journey away from the active synapse. Furthermore, both possibilities would require a large amount of protein to be seeded nearby potentially active synapses. A third alternative rests on the notion that the interactive properties between synapses expressing synaptic plasticity rely on dendritic protein synthesis [57] (conjectured in [36], [47]). In this scenario, activation of protein synthesis via plastic mechanisms could stretch to a single branch or a larger portion of a dendritic tree depending on the strength of the propagating signal originated in the active synapse(s). Hence, synaptic activation in a given dendritic region would set off the synthesis of specific protein factor(s) that would favor the expression of a particular form of synaptic plasticity within that region (functional compartment) [36].

### Transcompartmental LTP/LTD interference

Our data suggest that signals from each form of synaptic plasticity could meet and interfere with each other when generated at separate dendritic compartments, but such interference does not appear to require the synthesis of mRNA or protein. Evidence of pre-transcriptional mechanisms regulating gene expression at the level of activation of transcriptional activators and repressors could probably underlie transcompartmental LTP/LTD interference [59], [60], [61], [62], [63], [64].

Additionally, a mechanistic dissociation between intra and transcompartmental interactions might rely on changes in cellular excitability originated by strong synaptic activation. Changes in excitability can affect plastic mechanisms [65], and synaptically-driven dendritic depolarization can generate somatic spiking and spike back-propagation that can invade the opposing dendritic compartment [66] potentially modifying the expression properties of synaptic plasticity in that compartment. It is yet to be examined, however, whether such phenomenon occurs during LTP/LTD interference or whether synaptically-driven dendritic depolarization is restricted to just one dendritic compartment.

Can this compartmental restriction be overridden? It seems plausible to think that using a much stronger induction protocol for the induction of the prior (priming) form of synaptic plasticity would facilitate transcompartmental interaction. However, we have demonstrated that increasing the strength of the priming stimulation did not facilitate the cooperative interaction between a weak and a strong form of LTP across separate dendritic compartments [14].

### Simultaneous induction of LTP and LTD

When both forms of synaptic plasticity are induced simultaneously, LTD overpowers LTP within the same dendritic compartment, while LTP overpowers LTD across dendritic compartments. The precise time-interval dependent behavior of the LTP and LTD interactions breaks downs when both forms of plasticity are induced simultaneously. The nature of induction can regulate the outcome of the interaction between LTP and LTD [33], [44], [45], however, the nature of synaptic plasticity appears to be independent of LTP or LTD dominance during the interference between LTP and LTD. We are uncertain of the cause of these phenomena. If for instance, mechanisms for LTD induction “kick in” before LTP induction mechanisms, even if both inducing stimuli are delivered simultaneously, we would have seen always LTD overpowering LTP, irrespective of the location of the inputs. This is not the case in our study. To the best of our knowledge, we are unaware of any mechanism that could shed light into why one form of plasticity overpowers the other differently depending on the intra or transcompartmental location of the stimulated synaptic paths.

### Correlative behavior between interfering and cooperative LTP/LTD interactions

**Figure 7:**
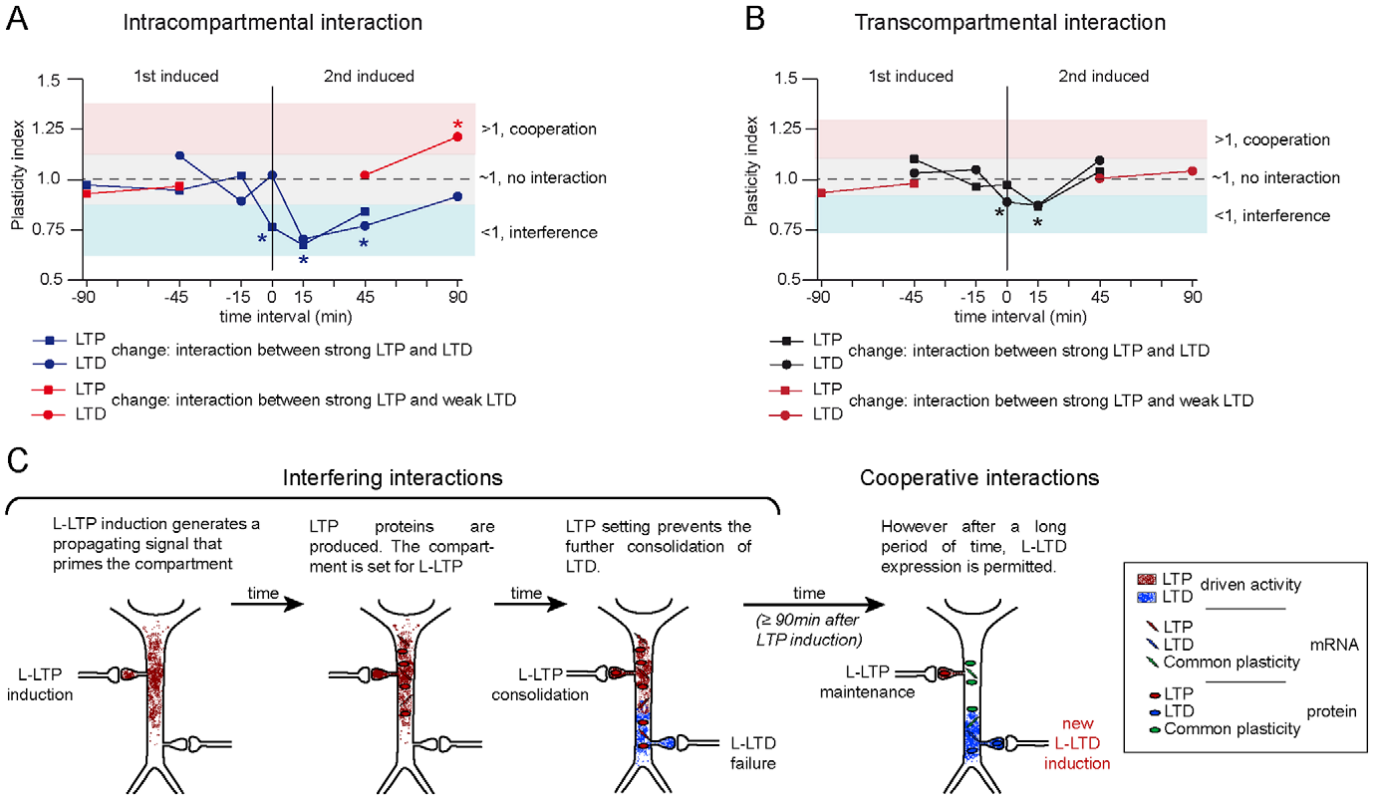
Correlated behavior of the interfering and cooperative interactions between LTP and LTD. (**A**): Graph representing the Plasticity Index (LTP change × LTD change for any given interaction) from all intracompartmental experiments carried out in this study. Despite the nature of the plasticity, the first form of plasticity remains largely unaffected (negative time intervals) compared to the second form of plasticity (positive time intervals). Remarkably, as interference between LTP and LTD wanes off (15, 45 and 90 min. time intervals, blue lines), cooperative interactions could be seen (45, 90 min time interval, red line). (**B**) Graph representing the Plasticity Index (LTP change × LTD change for any given interaction) from all transcompartmental experiments carried out in this study. Similar to intracompartmental interactions, irrespective of the nature of the plasticity, the first form of plasticity remains largely unaffected (negative time intervals) compared to the second form of plasticity (positive time intervals). Also similar to intracompartmental interactions, interference between LTP and LTD wanes off with the lengthening of the time interval between inductions (15 and 45 min. time intervals, blue lines), but no cooperative interactions were seen (45, 90 min time interval, red line) once interference subsided. Graphs in A and B highlight four main findings of our study: 1) that only the subsequent form of synaptic plasticity is affected, 2) that the strength of the interaction depends on the time separation between inductions, 3) that cooperation and interference can not occur at the same time intervals, and 4) that intracompartmental interactions are stronger in magnitude than transcompartmental ones. A fifth finding of our study is the dependency on new protein synthesis for the intracompartmental interference between LTP and LTD. **(C): Proposed model for the mechanistic switch from interfering to cooperative interactions between LTP and LTD within the same dendritic compartment.** We suggest that newly synthesized protein modulate the interaction between LTP and LTD in a temporally and spatially restricted fashion. The figure exemplifies the interaction between a strong form of LTP (L-LTP) and a strong form of LTD (L-LTD). We hypothesized that a limited amount of protein factors induced by either of these two types of synaptic plasticity would constrain other synapses localized within the same dendrites from consolidating an opposite form of synaptic plasticity. Once the activity of these protein factors has weakened, cooperative interactions (i.e. in this example capture of LTD) are allowed by the productively use of common plasticity proteins.

Our finding that the interactions between LTP and LTD within the same dendritic compartment are both spatially and temporally restricted suggests a common pathway between these two processes. Our study identifies a temporal restriction for the expression of interfering and cooperative interactions between LTP and LTD (Table 5 and figure 7A–B). Interference within the same dendritic compartment is observed with time intervals between inductions of 0, 15 and 45 min, but not with a 90 min interval (0 min and 15 min vs. 45 min in the case of transcompartmental interference). Remarkably, the cooperative interaction can be observed (only intracompartmentally) at the time interval which interference is no longer observed.

We propose a model (Fig. 7C) based on the compartment-specific capture of L-LTP [14] and on current notions for the integration of distinct forms of synaptic plasticity [15], [20], [36], [58]. Activity dependent mechanisms elicited by either strong LTP or LTD induction would prime a given compartment by modifying the availability of specific LTP or LTD factors (LTP or LTD mRNA and protein) to neighboring synapses. The synthesis of these protein factors would prevent synapses localized within the same functional dendritic compartment from consolidating an opposite form of synaptic plasticity. However, once the activity of these putative factors has subsided, cooperative interactions could be allowed by the productive use of all-purpose plasticity proteins, which are common for the expression of any form of synaptic plasticity.

### Functional compartmentalization

What is the size of a functional compartment? To gain insight into an estimation of the size of compartmentalization, we can address three issues, 1) the location of the active inputs, 2) the morphological distinction between each dendritic tree, and 3) the functional distinction between each dendritic tree. We know that the stimulated synaptic paths are input specific as paired-pulse facilitation (PPF) analysis shows no heterosynaptic cross-activation (see Methods, also demonstrated in [14]). This could ensure that at the synaptic level, we are activating two separate set of synapses. Each set of synapses belongs to either the same or different morphologically defined dendritic trees. In Alarcon et al. (2006) we demonstrated that synaptic tagging was restricted to each dendritic tree, suggesting a functional compartmentalization of the tag mechanism. Another independent study corroborated these findings and further characterized the molecular mechanisms of the tag in basilar and apical dendrites [20]. Altogether, we suggest that the functional size of the compartment would depend on the strength of the stimuli used for synaptic activation. Importantly, the key or operational word here is “functional”. Weak stimuli would generate a smaller functional compartment, while stronger stimuli a bigger one; having a single spine and the whole cell the lower and upper limit, respectively. Considering only the cable properties of a dendritic tree, the size of a functional compartment would not be defined by a metric function, but an activity one. The size of a functional compartment would be defined by a Gaussian-like activity function. It follows, therefore, that there would be no definitive boundaries for a given functional compartment, as plasticity-associated activity would peak at the site of the stimulated synapses and exponentially decay towards each side of the dendritic tree. This seemingly tidy depiction of a functional compartment changes if one considers mechanical barriers (e.g. soma, organelles) that would constrain the biochemical diffusion or transport of plasticity factors generated in response to synaptic activation. Then, how big would a functional compartment be in our studies? Here we argue that production of new protein is relevant for the intracompartmental interaction between LTP and LTD, and elaborate that the source of these proteins may be somatic or dendritic (nearby the site of synaptic activation). On the other hand, transcompartmental interaction is weaker than the intracompartmental one but not absent and appears independent of the synthesis of new protein. We think that the size of basilar and apical functional compartments extends and overlaps one to another at the level of electrotonic properties given that strong stimulus protocols as the ones used in this study would depolarize an entire dendritic tree and possibly invade the opposite tree [66]. But at the level of biochemical signals, namely protein factors, the size narrows down to match a morphologically defined dendritic tree. The size of the functional compartment might even narrow down to a dendritic domain (a fraction of the dendritic tree) if one considers the source of protein factors after synaptic activation to be only local (dendritic translation).

### Synaptic plasticity interactions and the encoding of information

Hippocampal neurons receive a large number of synaptic inputs potentially capable of inducing long-term synaptic plasticity. The cellular mechanisms regulating synaptic plasticity interactions (i.e. the interaction between synaptic inputs expressing synaptic plasticity in a single neuron) seem to be crucial for understanding the cellular basis of encoding multiplex information.

Encoding of information at specific compartments is mainly defined by the anatomy of hippocampal circuits [8], [37], [67]. Changes in neural activity that modulate hippocampal oscillations (e.g. theta, gamma) [68], [69] are suitable candidates to modulate the induction of synaptic plasticity in these synaptic paths [70], [71]. Indeed, changes in hippocampal oscillations occur with learning [72], [73], [74]. As induction of synaptic plasticity develops in various temporal fashions in multiple synapses, neurons will utilize synaptic plasticity interaction mechanisms to integrate these plastic events [14], [20], [35], [36]. Conceivably, the relationship between changes in input activity, hippocampal oscillations and synaptic plasticity interactions could impact a subset of the neuron population which could specifically encode multiplex information related to a given behavioral experience [75], [76], [77], [78]. Neurons within a particular population ensemble could therefore generate particular output spike activity stamps [79], [80], [81] that will impact the decoding of information in extra-hippocampal areas in order to produce behaviorally relevant outputs [82], [83], [84].

What could be the function of synaptic plasticity interactions? Particularly, how could the temporal and the spatial restrictions of the interaction between LTP and LTD lead to the proper encoding of information? Our study indicates that interactions between LTP and LTD in the CA1 area of the mouse hippocampus undergo spatial and temporal regulation. CA1 pyramidal neurons initially disfavor the coexistence of two opposite forms of synaptic plasticity. Dual existence (i.e. cooperative interactions) is only allowed after a given period of time. Interference between two inputs received at short time intervals provides a possible mechanism for disruption of unwanted information. Activity dependent disruption of unwanted information seems to be necessary for the stabilization of a memory trace [22], [85], [86], [87], [88]. In time, once a trace has been consolidated, another one can be associated to it.

Spatial and temporal interactions amongst plastic synapses of a neuron might enable the processing of information arriving into its distinct functional compartments from different brain areas, and associate or segregate such information. We propose that this information processing arises from the changes in synaptic weights due to synaptic plasticity interactions like those we have described.

## Materials and Methods

### Hippocampal Slice preparation

All procedures were approved by the Institutional Animal Care and Use Regulations of SUNY Downstate Medical Center. Animal Care and Use Committee (ACUC) protocol number: 12-347-09 and 12-347-10, Assurance Number: A3260-01. Adult (3–4 months old) male C57BL6 mice were transferred from the home caged to the anesthetizing induction chamber and remained in the covered chamber for 15–20 min for acclimation. Subsequently, animals were deeply anesthetized with 5% vaporized isoflurane in oxygen (100%) for 2–3 minutes, until absence of motility and ocular reflex. Animals were next euthanized via decapitation, the brain removed and placed into ice cold *dissection* **a**rtificial **c**erebrospinal **f**luid (*d*ACSF, containing in mM: 125 NaCl, 2.5 KCl, 7 MgSO_4_, 0.5 CaCl_2_, 25 NaHCO_3_, 1.25 NaH_2_PO_4_ and 25 Glucose) oxygenated with a 95%O_2_/5%CO_2_ mixture (ACSF pH 7.3). Isolated hippocampi were transversally sliced with a manual tissue chopper (MyNeurolab.com, USA) and dorsal transverse hippocampal slices (400 µm) were placed in an interface chamber (Campden Instruments, 745 Series), subfused with oxygenated *recording* ACSF (*r*ACSF: same as *d*ACSF but 1 MgSO_4_, 2 CaCl_2_; flow rate: 4 ml/min) at 34–35°C, and allowed to equilibrate for at least 120 min.

### Electrophysiology

Field excitatory postsynaptic potentials (fEPSP) from CA1 pyramidal cells were recorded from either apical or basal dendritic compartments by placing both stimulating (bipolar electrode #MX211ES, FHC & Co, USA) and recording (BF150-86-10 micropipette filled with *r*ACSF; pipette resistance 5–10 MΩ; Sutter Inst., USA) electrodes in the stratum radiatum or stratum oriens of the CA1 area, respectively. Before every experiment, synaptic input/output curves were generated and the stimulation intensity was adjusted to give fEPSP slopes of approximately 40% of maximum. Baseline, during and after stimuli responses were sampled once per minute at this intensity (test pulse, 50 µsec). Dual recording within the apical dendritic compartment or across the basal and apical dendritic compartments were performed as previously described [14], [47]. Briefly, by placing one pair of stimulating and one pair of recording electrode within the stratum radiatum we performed simultaneous recording from the apical dendritic compartments. By placing one stimulating and one recording electrode in the stratum oriens and another pair in the stratum radiatum we performed simultaneous recording from the basal and apical dendritic compartments. Both recording electrodes tips formed a vertical line that was perpendicular to the line formed by the stratum pyramidale. For each set of dual recording experiments we ensured pathway independency by testing paired-pulse facilitation [14], [47], [89] between afferents within the apical dendritic area and across dendritic compartments. We used anisomycin (20 µM, dissolved in ultra-pure water; Calbiochem, La Jolla, CA, USA) to block the synthesis of proteins and actinomycin-D (40 µM, dissolved in DMSO; Calbiochem, La Jolla, CA, USA) to block the synthesis of RNA. Aliquot of DMSO or ultra-pure water were used as vehicle in control experiments. The final concentration of DMSO was no higher than 0.05% (DMSO/rACSF). To rule out a potential direct effect of anisomycin or actinomycin over the subsequent expression of LTD, we pretreated (40 min) slices with each drug and 15 min later we induced LTD. This manipulation did not affect the expression of LTD (not shown).

### Statistical analysis

ANOVA analysis and Student's t test were performed using the Microcal Origin statistical tool (Microcal Software Inc. Northampton, MA, USA). For each set of drug-treated experiments independent vehicle-control experiments were done. In the text **n** represents the number of animals. For clarity in some figures the induction phase of LTD is not shown. In tables, controls and vehicle conditions are shown as pooled data; however, all statistical analyses are referred to the corresponding control or vehicle condition. Data in the text were presented as mean ± SD, whereas in figures as mean ± SE. The difference between the experimental data was considered significant at p<0.05.

### LTP and LTD change index

In Fig. 2, 3, 4 and 7, the LTP change was calculated as the ratio: LTP_120–150 min interaction_/LTP_120–150 min control_ where LTP_120–150 min_ is the relative change in fEPSP amplitude with respect to baseline between 120 and 150 min after LTP induction (as the values in the Tables). The LTD change was calculated as the ratio: 1/(LTD_120–150 min interaction_/LTD_120–150 min control_) where LTD_120–150 min_ is the relative change in fEPSP amplitude with respect to baseline between 120 and 150 min after LTD induction. We choose the invert ratio for the LTD change in order to have, when the interaction is interfering, both LTP and LTD changes smaller than 1, and when the interaction is cooperative, both LTP and LTD changes bigger than 1. Index values were considered different from 1 (1 = no interaction) when the original mean values for control and interaction conditions showed statistical difference (t-test, p<0.05).

## Supporting Information

Figure S1 **Severe interference between strong forms of LTP and LTD within the same dendritic compartment.** The representative traces from Fig. 2 are shown enlarged (gray: control; dark gray: interaction; 1: baseline, 2 after synaptic plasticity induction). Scale bar is 2 mV and 5 msec.
(TIF)

Figure S2 **Mild interference between strong forms of LTP and LTD across dendritic compartments.** The representative traces from Fig. 3 are shown enlarged (gray: control; dark gray: interaction; 1: baseline, 2 after synaptic plasticity induction). Scale bar is 2 mV and 5 msec.
(TIF)

Table S1 Intracompartmental interaction between strong forms of LTP and LTD in the apical dendritic compartment at early onset.
(DOC)

Table S2 Transcompartmental interaction between strong forms of LTP and LTD (LTP and LTD are induced at apical and basal dendrites, respectively) at early onset.
(DOC)
