# Supplementary figures and images for: Interaction between Long-Term Potentiation and Depression in CA1 Synapses: Temporal Constrains, Functional Compartmentalization and Protein Synthesis
Source: PLoS One. 2012 Jan 17;7(1):e29865. doi: 10.1371/journal.pone.0029865 (PMC3260185; doi:10.1371/journal.pone.0029865)

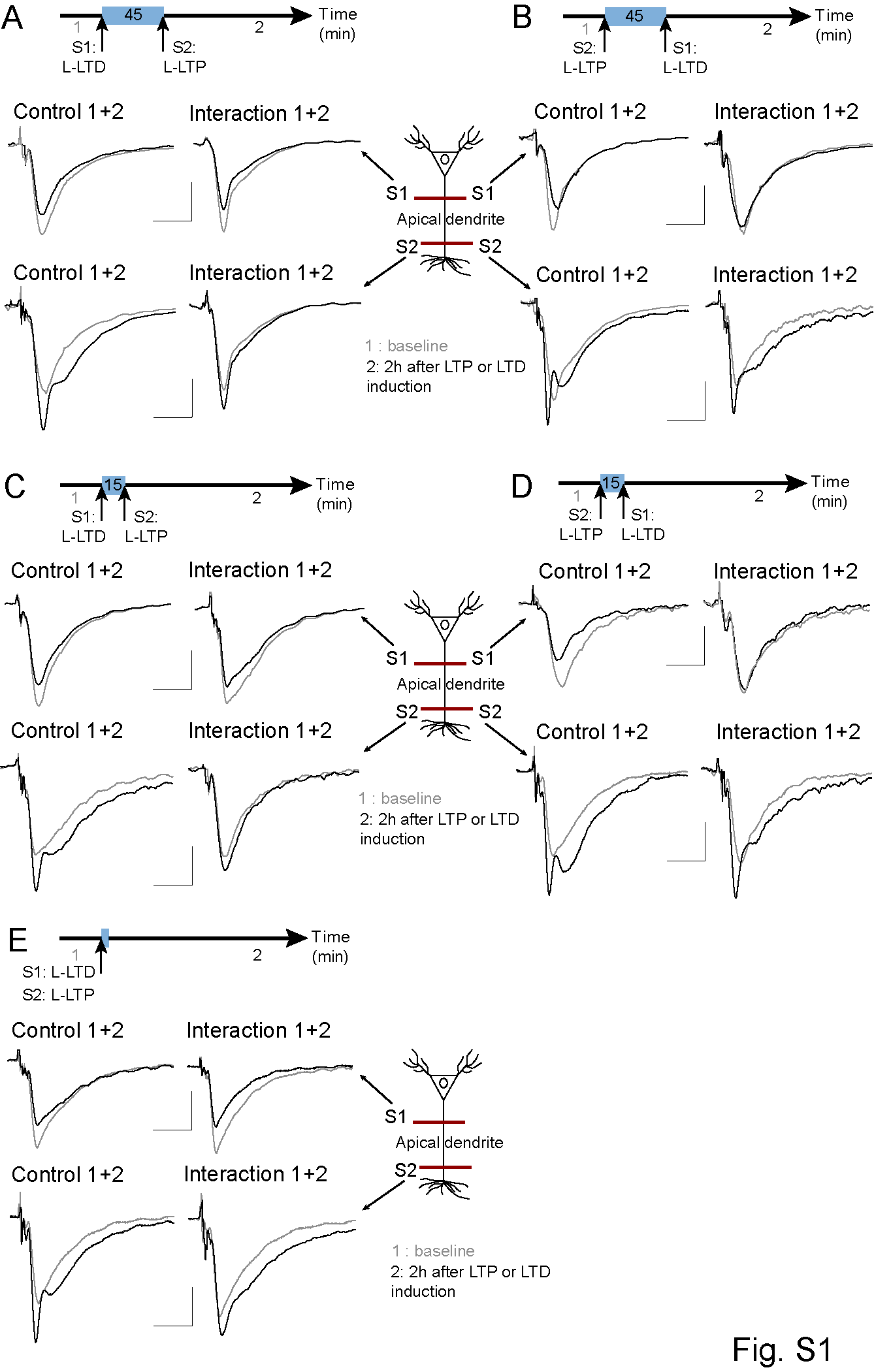

Supplement: Figure S1 — Severe interference between strong forms of LTP and LTD within the same dendritic compartment. The representative traces from Fig. 2 are shown enlarged (gray: control; dark gray: interaction; 1: baseline, 2 after synaptic plasticity induction). Scale bar is 2 mV and 5 msec. (TIF) [file pone.0029865.s001.tif]

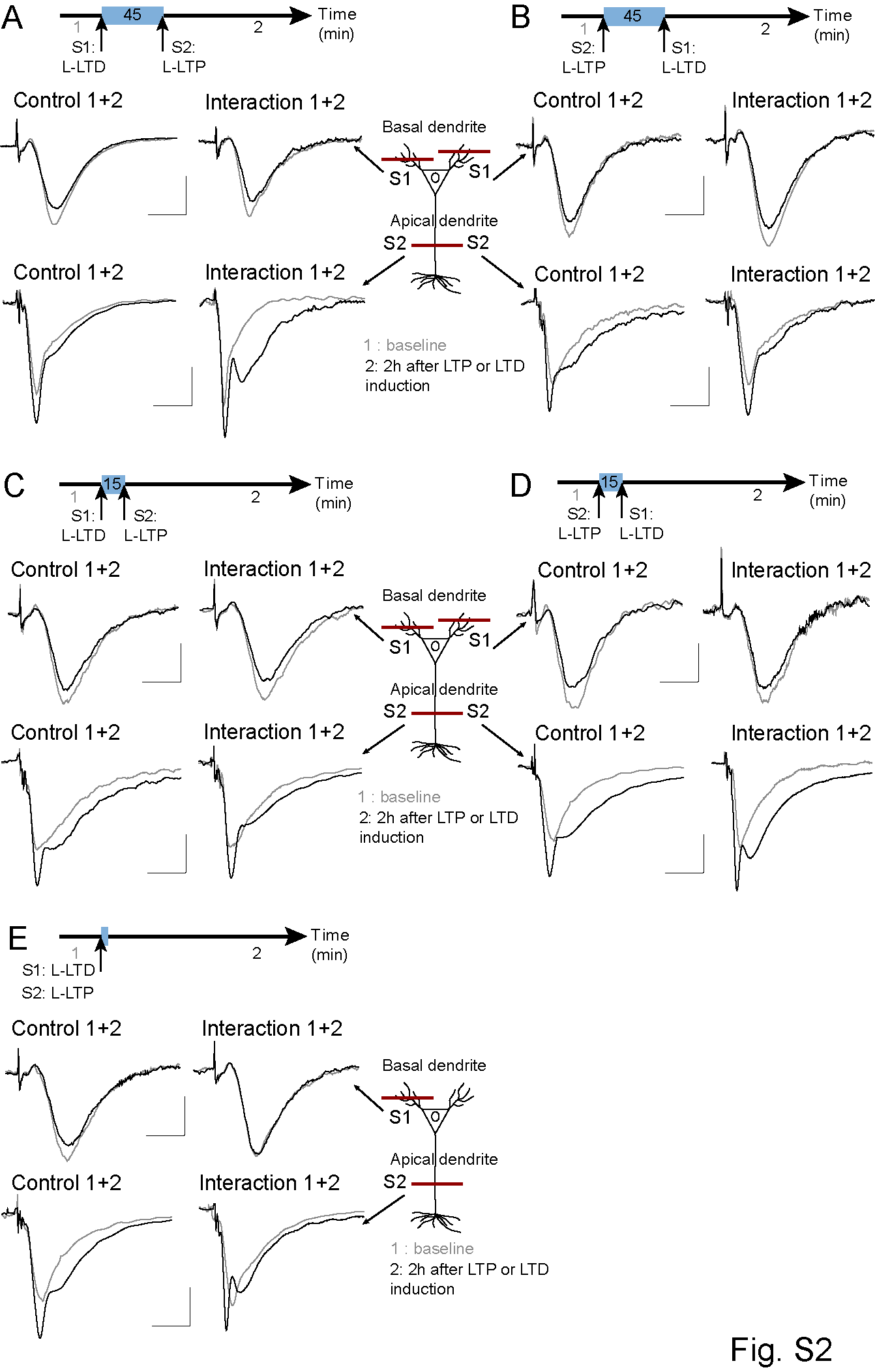

Supplement: Figure S2 — Mild interference between strong forms of LTP and LTD across dendritic compartments. The representative traces from Fig. 3 are shown enlarged (gray: control; dark gray: interaction; 1: baseline, 2 after synaptic plasticity induction). Scale bar is 2 mV and 5 msec. (TIF) [file pone.0029865.s002.tif]
