# Supplementary material for: Interaction between Long-Term Potentiation and Depression in CA1 Synapses: Temporal Constrains, Functional Compartmentalization and Protein Synthesis
Source: PLoS One. 2012 Jan 17;7(1):e29865. doi: 10.1371/journal.pone.0029865 (PMC3260185; doi:10.1371/journal.pone.0029865)
Supplement: Table S1 — Intracompartmental interaction between strong forms of LTP and LTD in the apical dendritic compartment at early onset. (DOC) [file pone.0029865.s003.doc]

**Table S1:** Intracompartmental interaction between strong forms of LTP and LTD in the apical dendritic compartment at early onset.

|  | **LTP 30-60 min** | **LTD 30-60 min** |
| --- | --- | --- |
| *CONTROL* | *199±9%* | *64±6%* |
| LTD before LTP 45’ | **149±6% *** | 71±4% |
| LTD before LTP 15’ | **145±10% *** | 67±8% |
| LTD & LTP | **179±13% *** | 63±4 % |
| LTP before LTD 15’ | 194±5% | **93±3% *** |
| LTP before LTD 45’ | 188±4% | **95±3% *** |

The values represent the relative change in fEPSP amplitude with respect to the baseline (100%).

***** Statistically significant from control at p<0.05
