# Supplementary material for: Interaction between Long-Term Potentiation and Depression in CA1 Synapses: Temporal Constrains, Functional Compartmentalization and Protein Synthesis
Source: PLoS One. 2012 Jan 17;7(1):e29865. doi: 10.1371/journal.pone.0029865 (PMC3260185; doi:10.1371/journal.pone.0029865)
Supplement: Table S2 — Transcompartmental interaction between strong forms of LTP and LTD (LTP and LTD are induced at apical and basal dendrites, respectively) at early onset. (DOC) [file pone.0029865.s004.doc]

**Table S2:** Transcompartmental interaction between strong forms of LTP and LTD (LTP and LTD are induced at apical and basal dendrites, respectively) at early onset.

|  | **LTP apical 30-60 min** | **LTD basal 30-60 min** |
| --- | --- | --- |
| *CONTROL* | *195±2.3 %* | *73±8.5 %* |
| LTD before LTP 45’ | 191±5.8% | 78±3 % |
| LTD before LTP 15’ | 184±10% | 78±3.7 % |
| LTD & LTP | 193±9.2 % | 90±5.1 % |
| LTP before LTD 15’ | 193±2.9 % | 80±3.3 % |
| LTP before LTD 45’ | 196±7.5 % | 72±2.7 % |

The values represent the relative change in fEPSP amplitude with respect to the baseline (100%)
